# Evaluation of Sperm DNA Fragmentation Using Two Different Methods: TUNEL via Fluorescence Microscopy, and Flow Cytometry

**DOI:** 10.3390/medicina59071313

**Published:** 2023-07-15

**Authors:** Katerina Chatzimeletiou, Alexandra Fleva, Theodoros-Thomas Nikolopoulos, Maria Markopoulou, Glykeria Zervakakou, Kyriakos Papanikolaou, George Anifandis, Anastasia Gianakou, Grigoris Grimbizis

**Affiliations:** 1 Unit for Human Reproduction, 1st Department of Obstetrics & Gynaecology, ‘Papageorgiou’ General Hospital, Aristotle University Medical School, 56403 Thessaloniki, Greece; thenikdim@auth.gr (T.-T.N.); grigoris.grimbizis@gmail.com (G.G.); 2 Department of Immunology and Histocompatibility, ‘Papageorgiou’ General Hospital, 56403 Thessaloniki, Greece; alexfleva@gmail.com (A.F.); mariamarkop@hotmail.com (M.M.); giannakouan@gmail.com (A.G.); 3 Fertilia by Genesis, IVF Unit, 54301 Thessaloniki, Greece; zervakakou@gmail.com (G.Z.); iatreiopap@outlook.com (K.P.); 4 Department of Obstetrics and Gynecology, School of Health Sciences, Faculty of Medicine, University of Thessaly, 41200 Larisa, Greece; ganif@med.uth.gr

**Keywords:** sperm, DNA fragmentation, tunel assay, flow cytometry, infertility

## Abstract

*Background and Objectives*: Sperm DNA fragmentation refers to any break in one or both of the strands of DNA in the head of a sperm. The most widely used methodologies for assessing sperm DNA fragmentation are the sperm chromatin structure assay (SCSA), the sperm chromatin dispersion assay (SCD), the single-cell gel electrophoresis assay (SCGE–comet), and the terminal-deoxynucleotidyl-transferase (TdT)-mediated dUTP nick end labelling (TUNEL) assay. The aim of this study was to compare the efficiency and sensitivity of the analysis of sperm DNA fragmentation using TUNEL via fluorescence microscopy, and flow cytometry. *Materials and Methods*: Semen samples were collected and analyzed for standard characteristics using light microscopy, and for sperm DNA fragmentation using both TUNEL via fluorescence microscopy, and flow cytometry. *Results:* There were no significant differences in the values of the sperm DNA fragmentation index (DFI) obtained when the analysis was performed using TUNEL or flow cytometry (*p* = 0.543). Spearman’s correlation analysis revealed a significant negative correlation between sperm motility (%) and sperm DNA fragmentation (*p* < 0.01), as well as between sperm concentration and sperm DNA fragmentation (*p* < 0.05). The Mann–Whitney U test showed no significant difference in the DFI among couples with repeated implantation failure (RIF) and miscarriages (*p* = 0.352). *Conclusions:* Both methods (TUNEL via fluorescence microscopy, and flow cytometry) have a high efficiency and sensitivity in accurately detecting sperm DNA fragmentation, and can be effectively used to assess male fertility.

## 1. Introduction

One in six couples is affected by infertility and, in a third of these cases, the cause is of male origin [1,2,3,4,5,6,7]. The most common way to evaluate male fertility is standard semen analysis, according to the WHO guidelines [8]. Nevertheless, subtle sperm defects, such as breaks in the DNA, cannot be identified using a standard semen analysis. Since the spermatozoon must deliver an intact genome for normal fertilization, the initiation of cleavage, and normal development, it is possible that DNA defects may influence reproductive outcomes [9,10]. High levels of sperm DNA fragmentation have been associated with lower fertilization rates, poor embryo quality, and delayed cleavage [11].

Sperm DNA fragmentation has not yet been shown to be a good predictor of positive HCG, clinical pregnancy, miscarriage, and live births during ICSI or IVF cycles [12]. A retrospective analysis of clinical outcomes in vitrified–warmed single-blastocyst transfer cycles, in relation to sperm DNA fragmentation, revealed no straight correlation between a positive hCG rate, a clinical pregnancy rate, and first trimester miscarriage, and DFI levels, suggesting that ART outcomes are not affected by sperm DNA fragmentation independently of the gamete quality [13]. A recent systematic review and meta-analysis on the impact of a very short abstinence period on sperm parameters and DNA fragmentation showed a significant increase in sperm concentration and motility in the second ejaculation, and a significant decrease in sperm DNA fragmentation [14]. A second consecutive ejaculation after a very short time from the first one could therefore be an easy and effective strategy for collecting better-quality spermatozoa.

Much attention has also been given to the assay used to evaluate sperm DNA fragmentation. The most widely used methodologies are the sperm chromatin structure assay (SCSA), the sperm chromatin dispersion assay (SCD), the single-cell gel electrophoresis assay (SCGE–comet), and the terminal-deoxynucleotidyl-transferase (TdT)-mediated dUTP nick end labelling (TUNEL) assay [15,16,17,18,19,20,21,22,23]. All of these tests are commercially available, but have different levels of sensitivity and specificity. There are studies that use either one assay or the other, jeopardizing the reliability of the results of other researchers who use different sperm DNA fragmentation tests. Even in large meta-analyses, the inclusion of studies with different sperm DNA fragmentation assays presents an obstacle to drawing solid conclusions [24].

The terminal-deoxynucleotidyl-transferase (TdT)-mediated dUTP nick end labelling (TUNEL) assay is one of the most reliable and sensitive methods for evaluating sperm DNA fragmentation [25,26]. The enzyme TdT is used to add labelled nucleotides to free 3′ OH ends of DNA strands, resulting in single-strand poly-U extensions. It can simultaneously detect single- and double-strand breaks, which constitutes an advantage of the test, and it can be performed via either fluorescence microscopy or flow cytometry [16,17,18,19,25,26]. In general, flow cytometry has been characterized as an automated, rapid, and sensitive method for evaluating male infertility [27,28,29]. Due to its multiparametric capability, it can be used to measure concentration in sperm samples, while the incorporation of DNA dyes differentiates haploid round spermatids and diploid cells from actually haploid mature spermatozoa. At the same time, it assesses spermatogenesis, motility, and viability [27,28,29]. Propidium iodide (PI), in combination with carboxyfluorescein-diacetate succinimidylester and SYBR-14 constitute the most common viability stains, which enter the spermatozoa emitting red or green fluorescence, respectively [17,18]. The aim of this study was to compare the efficiency and sensitivity of sperm DNA fragmentation analyses using TUNEL via fluorescence microscopy and flow cytometry.

## 2. Materials and Methods

Semen samples were collected and analyzed for standard characteristics using light microscopy at Fertilia by Genesis, Thessaloniki, Greece. The sperm DNA fragmentation was assessed using TUNEL, both via fluorescence microscopy, and via flow cytometry, at the Genetics Unit and the Department of Immunology and Histocompatibility at Papageorgiou Hospital, Thessaloniki, Greece. This study was approved by the Bioethics Committee of the Aristotle University Medical School (1.30/21 November 2018) and Genesis (01/7-2/3056). All analyses were performed following the patients’ informed consent.

### 2.1. Standard Semen Analysis

The semen analysis was performed according to the World Health Organization (WHO) criteria. The lower reference limits were: for volume, 1.5 mL; for concentration, 15 millions/mL; for progressive motility A + B, 32%; and for normal morphology, 4% [8].

### 2.2. Terminal Deoxynucleotidyl Transferase dUTP Nick end Labeling (TUNEL) via Fluorescence Microscopy

The evaluation of sperm DNA fragmentation using terminal deoxynucleotidyl transferase dUTP nick end labeling (TUNEL) via fluorescence microscopy was performed according to Chatzimeletiou et al. [19,30]. Ten microliters of fixed sperm suspensions in 3: 1 methanol: acetic acid (Sigma-Aldrich, Taufkirchen, Germany) were spread on polysine slides (Gerhard Menzel Braunschweig, Germany–Thermo Fisher Scientific, Singapore), and incubated in 0.1 M Tris/DTT swelling solution (Sigma-Aldrich, Taufkirchen, Germany) for 30 min, then washed in phosphate-buffered saline (PBS) (Sigma-Aldrich, Taufkirchen, Germany) and H_2_O, primed with TdT buffer and CoCl_2_ (Roche Diagnostics GmbH, Mannheim, Germany), and incubated with TdT buffer, CoCl_2_TdT enzyme, and dUTP (Roche Diagnostics GmbH, Mannheim, Germany) for 60 min in the dark. The slides were then placed in stop buffer, and were washed twice in PBS (Sigma-Aldrich, Taufkirchen, Germany). After staining with Texas Red (Vector Laboratories, Burlingame, CA, USA), the slides were washed in PBS (Sigma-Aldrich, Taufkirchen, Germany), air dried, mounted in Vectarshield antifade medium with DAPI (4, 6-diamidino-2-phenylidole; Vector Laboratories, Burlingame, CA, USA) under a coverslip, and sealed with nail varnish. Fragmented sperm head nuclei were assessed via fluorescence microscopy, using the Zeiss Imager.Z1 fluorescence microscope, equipped with TRITC (red) and DAPI (blue) filters, and the images were captured using Isis software (Metasystems, Altlussheim, Germany) [19,30]. The total number of sperm analyzed per sample was 1000. All sperm were stained blue with DAPI, but the fragmented sperm were additionally stained red with Texas Red. The calculation of the sperm DNA fragmentation index (DFI) using TUNEL via fluorescence microscopy was conducted by dividing the number of fragmented sperm head nuclei (red-stained) by the total number of sperm head nuclei (DAPI-stained). DFI= Number of fragmented sperm/1000.

### 2.3. Flow Cytometry

The flow cytometry was performed according to Chatzimeletiou et al. [31]. An aliquot of 100 μL of sperm samples was washed in PBS (Sigma-Aldrich Taufkirchen, Germany), and then centrifuged for 5 min at 300 g. After the removal of the supernatant, the precipitant was incubated in TNE buffer (NaCl (0.15 Μ), Tris HCL (0.01 Μ), EDTA (0.0011 Μ) pH 7.4) (Bioline Scientific, Athens, Greece) and detergent solution (NaCl 0.15 Μ, TRITON X-100) (Bioline Scientific, Athens, Greece) for 5 min. Acridine orange (Bioline Scientific Athens Greece) was added, and a further 5 min incubation followed, in the dark. The samples were finally analyzed via flow cytometry (Beckman Coulter, FC 500, South Kraemer Boulevard Brea, CA, USA), separating the intact sperm (green) from the fragmented ones (red), based on the change in color due to the acridine orange inserted into the fragmented portion of the sperm. The separation of sperm based on the FS/SS characteristics and gating on the viable cells removed any unwanted events, and allowed the calculation of the sperm DNA fragmentation index (DFI) in the viable portion of the cells. The calculation of the DFI using flow cytometry was performed by dividing the red fluorescence by the total red and green fluorescence. The DFI = red fluorescence/total red and green fluorescence.

### 2.4. Statistical Analysis

The statistical analysis of the results was carried out using the SPSS version 28.0.1.0 statistical package for Windows (IBM, New York, NY, USA).The whole group of patients (Group A, N = 35) was subdivided into two sub-groups: patients with a DNA fragmentation index (DFI) below 30% (detected by both methods) were allocated to Group B (N = 25), whereas patients with a DFI above 30% (detected by both methods) were allocated to Group C (N = 10). The normality of the data was assessed using the Shapiro–Wilk test. The non-parametric Wilcoxon signed-rank test was used to compare the medians of the DFI values obtained using the TUNEL and flow cytometry methods in both Group A and Group C. A paired-samples *t*-test was used to compare the means of the DFI values obtained using the TUNEL and flow cytometry methods in Group B. The Mann–Whitney U test was used to determine whether there were significant differences in the DFI between the infertility cases associated with repeated implantation failure (RIF), and those associated with miscarriage. Spearman’s test was used for the correlation analysis between the sperm concentration and DFI, and the sperm motility (%) and DFI. For the correlation analysis, each patient’s DFI was calculated as the average of the values obtained using both the TUNEL and flow cytometry methods. The statistical significance was set at *p* < 0.05. Box and whisker plots, and scatter plots were generated using Microsoft Excel software (version 2302, Microsoft Corporation, Redmond, WA, USA).

## 3. Results

The standard semen analysis and sperm DNA fragmentation assessed using both TUNEL and flow cytometry are shown in Table 1.

The descriptive statistics of the various sperm parameters in the different groups of patients are shown in Table 2.

The association of different causes of infertility with sperm DNA fragmentation is shown in Table 3.

**Figure 1:**
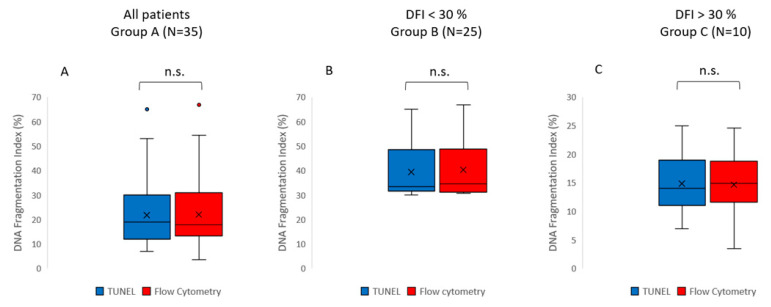
Box and whisker plots displaying the distribution of the data for sperm DNA fragmentation (DFI). The DFI values were obtained using either the TUNEL method (blue) or the flow cytometry method (red). (**A**) Group A includes all patients (N = 35); (**B**) Group B (N = 25) is a subgroup including the patients with DFI values below 30%, as detected by both methods; (**C**) Group C (N = 10) is a subgroup including the patients with DFI values above 30%, as detected by both methods. The box represents the interquartile range (IQR), which contains the middle 50% of the data. The line within the box represents the median. The whiskers extend from the top and bottom of the box to the minimum and maximum values within 1.5 times the IQR, respectively. Outliers are represented as individual points. The statistical analysis of all data (Group A) showed no significant difference in the DFI when assessed using TUNEL or flow cytometry (Wilcoxon signed rank test, *p* = 0.543). No significant differences in the DFI between TUNEL and flow cytometry were found in either Group B (paired samples *t*-test, *p* = 0.547) or Group C (Wilcoxon signed rank test, *p* = 0.169).

**Figure 2:**
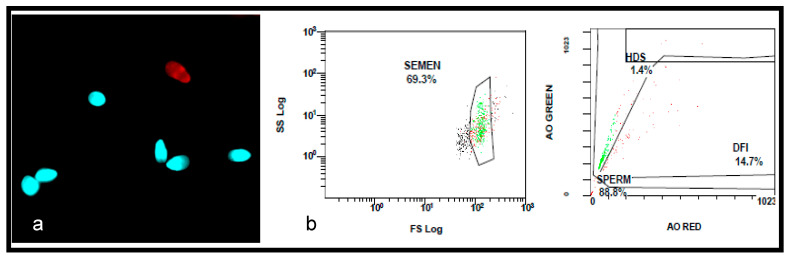
The assessment of sperm DNA fragmentation using TUNEL and flow cytometry. (**a**) A photomicrograph showing the TUNEL-labelled sperm. The normal spermatozoa are stained in blue with 4, 6-diamidino-2-phenylidole (DAPI), and the fragmented sperm is stained in red with Texas Red. The DFI was 14%. (**b**) The flow cytometry, showing 14.7% DFI.

**Figure 3:**
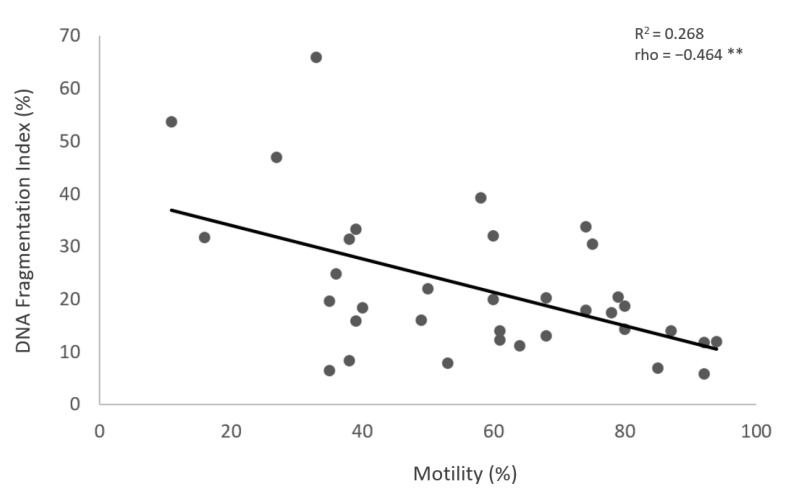
The correlation between the sperm motility (%) (X-axis) and the DFI (Y-axis). Individual data points and the regression line are shown. There is a significant negative correlation between the sperm motility (%) and the DFI. Spearman’s correlation coefficient (rho) = −0.464, *p*-value < 0.01. The notation (**) indicates that the *p*-value is less than 0.01.

**Figure 4:**
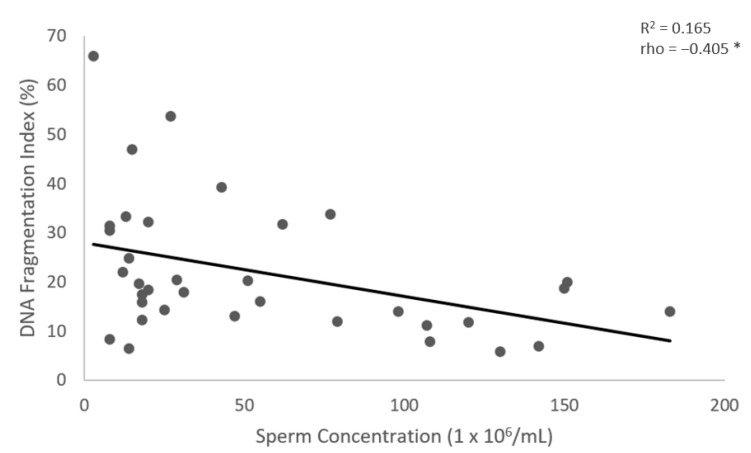
The correlation between the sperm concentration (X-axis) and the DFI (Y-axis). Individual data points and the regression line are shown. There is a significant negative correlation between the sperm concentration and the DFI. Spearman’s correlation coefficient (rho) = −0.405, *p*-value < 0.05. The notation (*) indicates that the *p*-value is less than 0.05.

The results show no significant differences in the values of sperm DNA fragmentation index (DFI) obtained when the analysis was performed using TUNEL or flow cytometry (*p* = 0.543) (Figure 1 and Figure 2, Table 2). Spearman’s correlation analysis revealed a significant negative correlation between sperm motility (%) and sperm DNA fragmentation (rho = −0.464, *p* < 0.01; Figure 3), as well as between sperm concentration and sperm DNA fragmentation (rho = −0.405, *p* < 0.05; Figure 4). The Mann–Whitney U test showed no significant difference in the DFI between couples with repeated implantation failure (RIF) and miscarriages (*p* = 0.352) (Table 3).

## 4. Discussion

Sperm DNA fragmentation refers to any break in one or both of the strands of DNA in the head of a sperm [20]. Breaks in the DNA may play a crucial role in gamete function and, as a consequence may affect fertilization, embryo development, and the reproductive outcome [9,10,11,12,13]. Aspects of sperm function that can be disrupted include motility and sperm–zona recognition. Sperm transcripts and proteins are involved in acrosome reaction and fusion and, once released into the oocyte, can influence embryo development. The impact DNA fragmentation may have on the success of assisted reproduction cycles highly depends on the balance between the extent of the DNA breaks, and the ability of the oocyte to repair this damage. This diversity in sperm DNA damage and the repair capacity of the oocyte may explain why some fragmented sperm retain their fertilizing ability [9,10,11,12,13,20,26].

The most widely used methodologies for assessing sperm DNA fragmentation are the sperm chromatin structure assay (SCSA), the sperm chromatin dispersion assay (SCD), the single-cell gel electrophoresis assay (SCGE–comet), and the terminal-deoxynucleotidyl-transferase (TdT)-mediated dUTP nick end labelling (TUNEL) assay [16,17,18,19,20,21,22,23]. Our study compared the efficiency and sensitivity of sperm DNA fragmentation analysis using TUNEL via fluorescence microscopy, and flow cytometry, and showed no significant differences in the values of the sperm DNA fragmentation index (DFI) obtained when analysis was performed using either of the two methods. Additionally, our study revealed a significant negative correlation between the sperm motility and sperm DNA fragmentation, as well as between the sperm concentration and sperm DNA fragmentation. Our results are in agreement with previous studies, confirming the reliability of both methods in assessing sperm DNA fragmentation [16,17,18,19,21,22,23,24,25,26,27,28,29,30,31]. The reliable measurement of sperm DNA fragmentation is of utmost importance to the identification of the causes of male infertility, and a deep understanding of the mechanisms leading to DNA fragmentation may provide new management strategies for overcoming male infertility.

Various mechanisms have been proposed as leading to sperm DNA fragmentation, including (a) the abortive apoptosis theory, (b) the defective maturation theory, and (c) oxidative stress [32,33,34,35,36]. The abortive apoptosis theory suggests that DNA fragmentation may originate in the testis, as part of the normal process of apoptosis, or as a consequence of different insults during transit in the genital tract, and that it is induced by activated endonucleases, which mostly lead to DNA double-stranded breaks [32,33]. According to this theory, fragmented sperm in the ejaculate may be derived from germinal cells whose apoptotic process was not completed in the testis [32,33]. On the other hand, the defective maturation theory suggests that DNA fragmentation may occur during chromatin compaction, as a result of histones’ replacement by protamines [34]. However, these two mechanisms cannot provide a full explanation for the occurrence of DNA fragmentation in the ejaculate, especially as higher levels of DNA fragmentation have been observed in the caudal epididymis and the ejaculate than in testicular sperm [35,36]. The generation of reactive oxygen species (oxidative stress) appears to be the main cause of DNA fragmentation, following release from the testis. Genitourinary infections, varicocele, and immature spermatozoa retaining cytoplasmic droplets may lead to excessive intrinsic reactive oxygen species production, increasing sperm DNA fragmentation [35,36].

The modern lifestyle may also predispose men to increased sperm DNA fragmentation [1,2,3]. Cigarette smoking, excessive alcohol consumption, an unhealthy diet leading to obesity which can, in turn, be linked to diabetes, a lack of exercise, exposure to environmental pollutants, an elevated testicular temperature from computers/laptops, hot tubs, and tight-fitting underwear are all contributing factors [1,2,3,6,37,38,39]. Sperm with fragmented DNA look normal in morphology, are motile and viable, and can successfully fertilize oocytes. However, embryonic development and subsequent implantation may be impaired in embryos derived from sperm with fragmented DNA. Although oocytes have the machinery to repair DNA damage, factors such as the quality of the oocyte itself and the type of sperm DNA damage may influence the extent to which this repair occurs [40,41,42]. Therefore, it is of utmost importance to accurately diagnose DNA fragmentation in sperm and, in men with high levels of sperm DNA fragmentation, suggest suitable treatments before the initiation of their assisted reproduction cycle. Given that new sperm are generated every 72 days, decreasing exposure to oxidative stress by making lifestyle changes, avoiding smoking, limiting alcohol intake, and incorporating a healthy diet and exercise, as well as considering the use of supplements containing vitamins and antioxidants, may decrease the degree of sperm DNA fragmentation [43,44]. Surgical repair, in cases of varicocele, may also be considered, if proved to be necessary [45,46].

In general, apoptosis and sperm chromatin maturation defects are believed to act in the testis, and cause the DNA breaks found in non-viable ejaculated spermatozoa [47,48]. On the other hand, oxidative stress induces sperm DNA fragmentation following release from the testis, during the transit through the male genital tract, and causes the DNA breaks found in viable spermatozoa in the ejaculate [47,48,49]. Oxidative stress is also suggested to be the main mechanism inducing sperm DNA fragmentation after ejaculation during in vitro manipulation [50]. All assisted reproductive technologies (ARTs), including intrauterine insemination (IUI), conventional in vitro fertilization (IVF), and intracytoplasmic sperm injection (ICSI), require the handling and micromanipulation of sperm. The most widely used methodologies for sperm selection during ART treatments are density-gradient centrifugation (DGC) and swim-up, which enable the selection of the most highly motile and morphologically normal spermatozoa [50,51,52]. Sperm may sustain damage both during the selection process, and when remaining for an extended time in the incubator before insemination. Spermatozoa may also acquire additional damage when selected with more advanced technologies, using high magnification (IMSI), as they remain exposed to light for longer before insemination [50]. Any damage of this type may alter sperm characteristics and functions. The motility, morphology, mitochondrial function, and ability to undergo the acrosome reaction may be altered, affecting fertilization rates, embryo quality, and subsequent embryonic development. Whether or not mature spermatozoa are able to trigger apoptotic pathways warrants further investigation [45,46,47,48,49].

In the current study, we compared the efficiency and sensitivity of sperm DNA fragmentation analysis using TUNEL via fluorescence microscopy, and flow cytometry. Our results showed no significant differences in the sperm DNA fragmentation index (DFI) values obtained when analysis was performed using TUNEL or flow cytometry and, additionally, revealed a significant negative correlation between the sperm motility and sperm DNA fragmentation, as well as between the sperm concentration and sperm DNA fragmentation. These results are in agreement with previous studies, confirming the reliability of both methods [16,17,18,19,21,22,23,24,25,26,27,28,29,30,31,45]. In contrast, the comet and SCD–HALO tests, which measure only a limited number of sperm (50–200) per sample, suffer from their lacking the statistical robustness of flow cytometric or TUNEL measurements [16,19,22,30,31,53].

## 5. Conclusions

We conclude that both methods used in this study (TUNEL via fluorescence microscopy, and flow cytometry) have a high level of efficiency and sensitivity in accurately detecting sperm DNA fragmentation. No statistically significant differences in sperm DNA fragmentation were obtained when the analysis was performed either using TUNEL or flow cytometry, and no straight correlation was observed amongst different couple’s indications, and DNA fragmentation. Larger-scale studies are needed, to elucidate any potential associations between fragmentation levels, repeated implantation failure (RIF), and miscarriages. The reliable measurement of sperm DNA fragmentation is of utmost importance in identifying the causes of male infertility, and a deep understanding of the mechanisms leading to DNA fragmentation may open new horizons for the therapeutic treatment of infertile males.

## Figures and Tables

**Table 1:** The sperm DNA fragmentation assessment using TUNEL and flow cytometry, and standard semen analysis. DFI, DNA fragmentation index; RIF, repeated implantation failure (lower reference limits: for concentration/mL, ≥15 × 10^6^; for motility A + B, ≥32%; for DFI, ≤29).

Sample Number	Concentration/mL ×10^6^	Motility A + B %	DFI Using TUNEL Fluorescence Microscopy %	DFI Using Flow Cytometry %	Couple’s Infertility
1	8	38	9	7.6	RIF
2	130	92	8	3.5	Miscarriages
3	150	80	19	18.4	Endometrial factor
4	62	16	32	31.3	Miscarriages
5	8	38	32	30.8	Biochemical Pregnancies
6	55	49	17	15.1	Miscarriages
7	151	60	19	20.8	RIF
8	120	92	11	12.5	Biochemical Pregnancies
9	79	94	12	12.0	Miscarriages
10	77	74	35	32.6	Biochemical Pregnancies
11	98	61	13	14.9	Unexplained
12	51	68	20	20.4	RIF
13	12	50	22	21.8	RIF
14	15	27	47	46.8	Miscarriages
15	14	36	25	24.6	Miscarriages
16	20	40	19	17.8	RIF
17	183	87	14	14.0	RIF
18	25	80	14	14.7	Miscarriages
19	31	74	18	17.8	Miscarriages
20	18	78	18	16.7	RIF
21	3	33	65	66.8	RIF
22	43	58	38	40.5	Miscarriages
23	17	35	20	19.1	Unexplained
24	18	39	15	16.6	RIF
25	8	75	30	31.0	Miscarriages
26	20	60	30	34.2	Miscarriages
27	13	39	32	34.7	Miscarriages
28	107	64	11	11.3	Endometrial factor
29	142	85	7	6.8	Endometrial factor
30	108	53	8	7.6	Endometrial factor
31	29	79	20	20.9	RIF
32	14	35	8	4.9	Endometrial factor
33	27	11	53	54.5	Miscarriages
34	18	61	12	12.5	Miscarriages
35	47	68	12	14.0	RIF

**Table 2:** The descriptive statistics of various sperm parameters, between the groups of patients. Groups B and C are sub-groups of Group A. The sperm DNA fragmentation (%DFI) was assessed using both TUNEL and flow cytometry.

		Group A (N = 35) All Patients			Group B (N = 25) Patients with DFI < 30%			Group C (N = 10) Patients with DFI > 30%		
Sperm Parameters		Mean ± S.E.M.		Median	Mean ± S.E.M.		Median	Mean ± S.E.M.		Median
Concentration (×10^6^/mL)		54.89 ± 8.68		29	65.8 ± 11.07		47	27.6 ± 7.93		17.5
Progressive Motile (×10^6^/mL)		38.94 ± 7.48		20	49.48 ± 9.51		27	12.6 ± 5.4		5.5
Progressive Motility (%)		57.97 ± 3.8		60	63.92 ± 3.96		64	43.1 ± 7.18		38.5
Non-Progressive Motile (×10^6^/mL)		4.06 ± 0.67		2	4.8 ± 0.87		2	2.2 ± 0.57		1.5
Immotile (×10^6^/mL)		11.89 ± 2.13		8	11.52 ± 2.38		8	12.8 ± 4.69		8
		Mean ± S.E.M.	Median	*p*-value	Mean ± S.E.M.	Median	*p*-value	Mean ± S.E.M.	Median	*p*-value
DFI (%)	TUNEL	21.86 ± 2.28	19		14.84 ± 1	14		39.4 ± 3.74	33.5	
	Flow Cytometry	21.99 ± 2.38	17.8	0.543	14.65 ± 1.1	14.9	0.547	40.32 ± 3.85	34.45	0.169

**Table 3:** The association of different causes of infertility with sperm DNA fragmentation.

Cause of Infertility	Number of Cases	%DFI (Mean ± S.E.M.)
Repeated Implantation Failures (RIF)	11	21.38 ± 4.61 ^a^
Miscarriages	14	26.47 ± 3.79 ^a^
Endometrial Factor	5	10.2 ± 2.28
Biochemical Pregnancies	3	25.65 ± 6.98
Unexplained	2	16.75 ± 2.8

^a^ *p*-value = 0.352 (Mann–Whitney U test).

## Data Availability

The data presented in this study are available on request from the corresponding author.
